# Muscle stem cells and rotator cuff injury

**DOI:** 10.1016/j.xrrt.2021.05.001

**Published:** 2021-05-21

**Authors:** Ranjan Gupta, Rohan Rao, Tyler R. Johnston, Jennifer Uong, Daniel S. Yang, Thay Q. Lee

**Affiliations:** a Department of Orthopaedics, University of California, Irvine, CA, USA; b Congress Medical Foundation, Pasadena, CA, USA

**Keywords:** Muscle stem cells (MuSCs), Rotator cuff tears (RCTs), Goutallier stages, Fatty infiltration, Adipogenic, Myogenic

## Abstract

The incidence of reinjury after treatment of rotator cuff tears (RCTs) remains very high despite the variety of nonoperative treatments and the high volume of surgical interventions performed. Muscle stem cells (MuSCs), also known as satellite cells, have risen to the forefront of rotator cuff tear research as a potential adjuvant therapy to aid unsatisfactory surgical outcomes. MuSCs are adult stem cells exhibiting the capacity to proliferate and self-renew, both symmetrically and asymmetrically. As part of this niche, they have been shown to adopt an activated phenotype in response to musculoskeletal injury and decrease their cellular populations during aging, implicating them as key players in both pathologic and normal physiological processes. While commonly connected to the regenerative phase of muscle healing, MuSCs also have the potential to differentiate into adverse morphologies. For instance, if MuSCs differentiate into adipocytes, the ensuing fatty infiltration serves as an obstacle to proper muscle healing and has been associated with the failure of surgical management of RCTs. With the potential to both harm and heal, we have identified MuSCs as a key player in RCT repair. To better understand this dichotomy, the following review will identify key studies regarding the morphology, function, and behavior of MuSCs with respect to RCTs and healing.

Approximately 30% of individuals older than 60 years of age have been shown to have a full-thickness rotator cuff tear (RCT) of at least one tendon.^33^ In patients older than 80 years of age, the likelihood of RCT further increases to as high as 80%.^76^ Although a variety of clinical treatments for RCTs exist, ranging from nonoperative physical therapy to surgical interventions, the eventual outcome of treatment may be unsatisfactory as results remain highly variable despite optimal medical management. Moreover, recurrence of injury after surgical repair involving 2 tendons has been reported to be as high as 41%, attributed to a variety factors, including muscle atrophy, retraction, and fatty infiltration.^22^ This combination of significant patient volumes and unsatisfactory surgical outcomes presents an opportunity for adjuvant treatment strategies to significantly ameliorate postrepair RCT deterioration.

To analyze current adjuvant treatments, the pathology of musculoskeletal injuries—and that of RCTs in particular—must first be detailed. Human skeletal muscle consists of myofibers, neurons, fibroblasts, adipose tissue, and connective tissue.^19^ Although there exists a heterogeneity of cell types, injuries to skeletal muscle mainly involve degeneration of myofibers.^59^ The loss of tendon attachment to bone substantially changes muscle physiology, structure, and function with the ensuing decreased muscle tensile forces also leading to decreased muscle strength. Without normal tensile loading forces, muscle atrophy ensues, both radially and longitudinally.^83^ Ultrastructurally, there is a decrease in sarcomere number and length, resulting in myofiber disorganization.^5^^,^^32^ At the macrolevel, it is believed that this decrease in muscle fibers and mass leads to increased fat content and fibrosis which can culminate in surgical complications, as will be discussed in the next section.^37^^,^^56^^,^^86^

## Fatty infiltration’s inhibition of satisfactory surgical outcomes

RCTs are believed to induce fatty infiltration, in addition to associated obligate muscle atrophy.^36^^,^^63^^,^^86^ This fatty infiltration poses a unique problem as surgical outcomes have been shown to decline in the presence of significant fatty infiltration.^26^^,^^53^ Muscle atrophy after RCTs is a principal concern for surgeons as even after controlling for muscle cross-sectional area, fatty infiltration is a predictor of supraspinatus weakness.^80^ Supraspinatus outcomes are also impacted by the fatty makeup of infraspinatus and subscapularis muscles, as fatty degeneration of these muscles has been shown to increase the likelihood of tear recurrence.^26^ Despite a clear association with substandard surgical outcomes, the cellular origins of fatty infiltration remain a critical gap in our understanding of rotator cuff pathophysiology.^47^ Many researchers have pointed to a variety of stem and progenitor cells as the source of fatty infiltration, with 2 major candidates emerging – PDGFRα+ progenitor cells and muscle stem cells (MuSCs).^51^^,^^73^^,^^79^ Lineage tracing experiments demonstrate that PDGFRα+ progenitor cells have the potential to become brown adipocytes or white adipocytes depending on the nature of inductive signals received.^44^^,^^46^ On the other hand, MuSCs also remain a possible source of adipocytes as a result of their multilineage potency.^3^^,^^66^^,^^70^

Clinically, Goutallier et al^26^ created a classification system based on computed tomography imaging to quantify the inherently qualitative nature of fatty infiltration, ranging from stage 0 (normal muscle) to stage 4 (more fat than muscle); this has subsequently been adapted to magnetic resonance imaging as well.^74^ Higher stages reported by Goutallier et al correspond to more severe RCT fatty infiltration. Previously, Fuchs et al^21^ verified that magnetic resonance imaging is a reproducible method for evaluating and staging fatty degeneration severity. The stages reported by Goutallier et al are particularly relevant to rotator cuff repair as stages reported by Goutallier et al greater than 2 have been shown to have lower surgical success rates.^24^ As of yet, no study has reported a link between stem or progenitor populations and stages reported by Goutallier et al, which raises the question of whether MuSC populations are being preferentially differentiated to an adipocyte phenotype in advancing stages reported by Goutallier et al.

## Morphology and function of MuSCs

**Figure 1:**
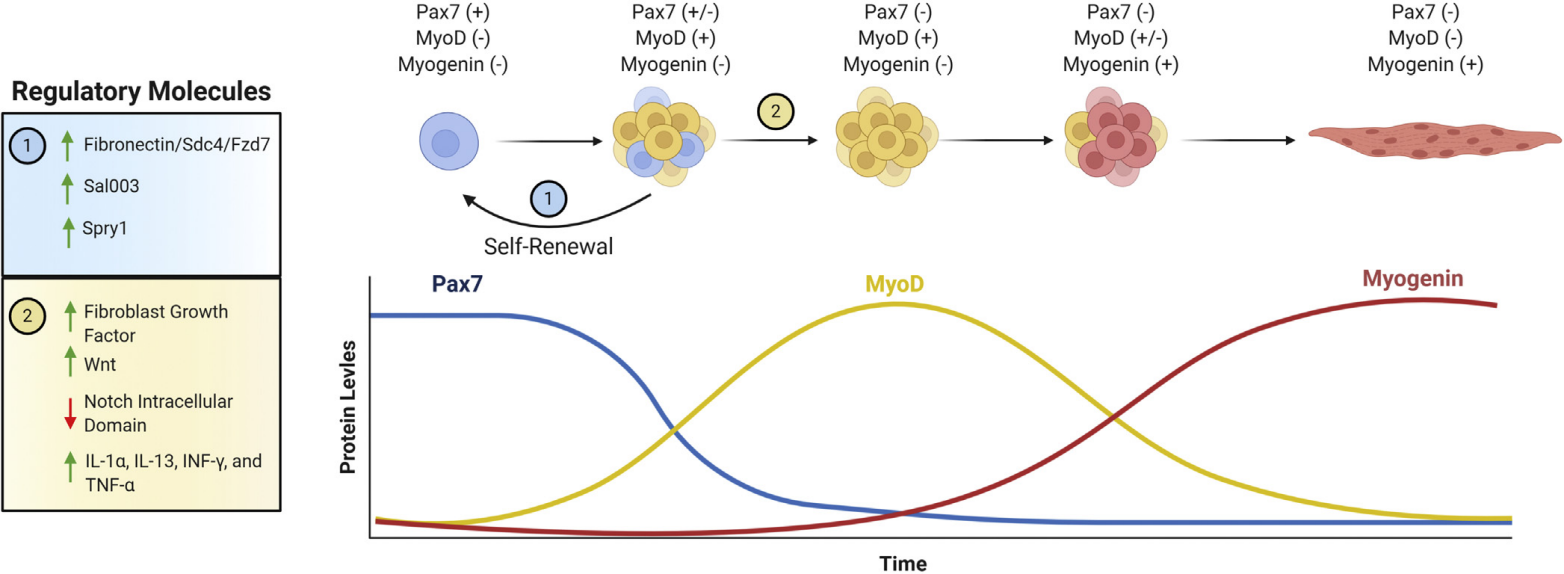
Overview of the distinct protein marker profiles and regulatory molecules involved in the progression of MuSCs down the myogenic fate. *MuSC*, muscle stem cell.

MuSCs were first discovered in 1961 when Mauro^52^ detected a clump of mononucleated cells in adult myofiber using electron microscopy. Physically, MuSCs reside between the basal lamina and sarcolemma of adult muscle fibers.^2^^,^^17^ They display the typical characteristics of adult stem cells in that they possess the ability to proliferate and self-renew. MuSCs are identifiable by their unique positive marker profile that includes Pax7/Pax3, CXCR4, CD56, and CD29.^10^^,^^40^^,^^81^ Pax3 and Pax7 are transcription factors that regulate myogenic differentiation of MuSCs by activating the expression of myogenic differentiation genes *Myf5* and *MyoD*.^37^^,^^40^^,^^62^ CXCR4, or C-X-C chemokine receptor type 4, is known for its role in regulation of cell migration and is highly expressed in MuSCs.^8^^,^^61^ CD56, or neural cell adhesion molecule, is expressed in human MuSCs but not in mouse MuSCs.^10^^,^^13^^,^^79^ And finally, CD29, also known as integrin beta-1, functions mainly as a collagen receptor but has been shown to be upregulated in MuSCs.^35^ Consequently, a mature, quiescent MuSC has a marker profile of Pax7^+^/MyoD^-^/Myogenin^-^, whereas a myogenic precursor will display Pax7^-^/MyoD^+^/Myogenin^-^, and the final myotube will exhibit Pax7^-^/MyoD^-^/Myogenin^+^.^16^ Using these marker profiles, the progression of MuSCs can be tracked through their life cycle (Fig. 1). Furthermore, marker identification of human MuSCs in pathologic rotator cuff muscles has significant utility for the detection and investigation of normal vs. abnormal myogenic differentiation after RTC.

Proliferating human and murine MuSCs have been shown to be capable of myogenic, adipogenic, and fibrogenic differentiation *in vitro* and *in vivo*.^9^^,^^11^^,^^58^^,^^60^^,^^67^^,^^75^^,^^84^ This multilineage differentiation capability sheds light on the possible double-edged contribution of MuSCs in both muscle regeneration and pathogenesis. On one hand, MuSCs display a remarkable myogenic regenerative capacity as Collins et al^15^ showed that as few as 7 MuSCs from a single transplanted muscle fiber could develop into more than 100 new myofibers. On the contrary, growing evidence of MuSC’s adipogenic and fibrogenic potential suggests their pathological capacity and role in muscle degeneration. A recent *in vitro* study demonstrated the reduced myogenic and increased adipogenic differentiation capacity of mouse rotator cuff MuSCs compared with that of mouse gastrocnemius MuSCs suggesting an underlying cellular and genetic basis unique to rotator cuff MuSCs behind the fatty degeneration observed in RCTs.^67^ Finally, the fibrogenic potential of MuSCs has been shown in a mouse model of Duchenne muscular dystrophy where *in vivo* lineage tracing revealed that a fraction of MuSCs in *mdx* mice had lost their myogenic fate and displayed a fibrogenic phenotype with increased expression of fibrotic genes.^9^ While the multilineage capability of MuSCs provides versatility, it also allows for MuSCs to differentiate into cells such as fibroblasts and adipocytes which are inherently detrimental to muscle healing.^39^ Manipulation of these undesired differentiation pathways has already been attempted by multiple researchers and represents a possible future direction for therapeutics directed at modulating MuSC niche signals for the treatment of musculoskeletal injuries and diseases. For example, Biressi et al showed that the pharmacologic inhibition of the TGFβ pathway *in vivo* inhibited MuSC fate change in *mdx* mice toward fibrogenic cell differentiation.^9^ Further possible clinical applications that warrant investigation in large animal models first may include the administration of exosomes or growth factors so as to foster and ensure differentiation of MuSC into a myogenic lineage as an adjunct to RTC repair surgery.

Muscle injury is the most common condition in which MuSCs proliferate. Upon injury, MuSCs exit their quiescent state and transform to committed progenitor cells called myoblasts, which then fuse with each other as well as injured myofibers.^65^^,^^82^ It has been hypothesized that the muscle atrophy found in injury states is attributable to the decrease in MuSC proliferation, number, and ultimately differentiation.^34^ Biopsies from patients undergoing arthroscopic rotator cuff surgery found that while the MuSC population was larger in muscles from cuffs with partial tears compared with no tear or full-thickness tears, these MuSCs had reduced proliferative ability.^54^ Similarly, Thomas et al found ^77^a 100-fold slower proliferation capacity in MuSCs from patients with partial- or full-thickness tears compared with MuSCs from patients with no tear. Furthermore, human RCT muscle tissue is distinctly pathologic, with proteomic analysis revealing increased extracellular matrix deposition and a shift in muscle composition in the pathologic state.^77^

While injury is the more common instigator of MuSC differentiation, aging has also been shown to deplete multiple stem and progenitor populations, causing individual stem cells and progenitors to activate in response to tissue loss or other changes. This pattern holds true in MuSCs where increased levels of fibroblast growth factor (FGF), highly expressed in aged murines, lead to loss of quiescence and diminishment of the murine MuSC niche.^14^ Therefore, aging leads to upregulation of FGF, which in turn diminishes the MuSC niche.^14^ Although there are currently no clinical studies focused on this arena, FGF blockage is an important target for adjuvant therapy that warrants further investigation based on these animal studies. As the likelihood of RCT increases with age,^76^ the relationship among age, diminishing MuSC niche, and increasing RCT frequency may be another clinically relevant area of study.

**Figure 2:**
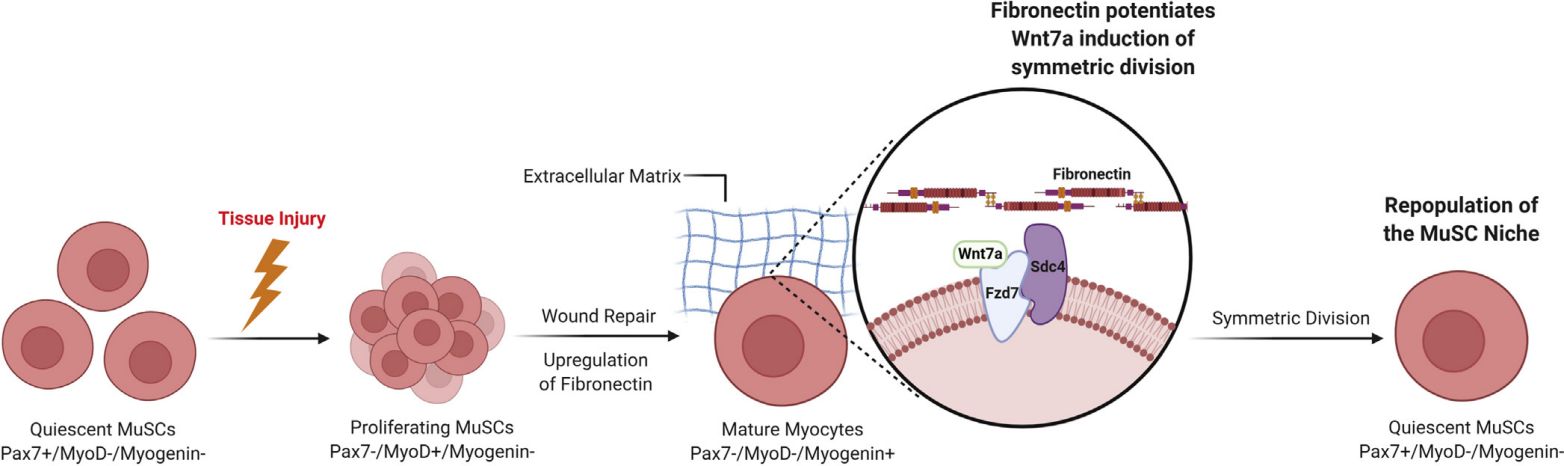
Mechanism of MuSC niche self-renewal and maintenance through symmetric division. *MuSC*, muscle stem cell.

MuSCs self-renew primarily through either asymmetric or symmetric self-renewal.^52^^,^^82^ Symmetric self-renewal is the prototypical mitotic division in which 2 identical daughter cells are produced, subsequently reoccupying the MuSC niche (Fig. 2). Symmetric expansion is regulated through the cell surface coreceptor complex of Syndecan-4 and Frizzled-7, which stimulates the ability of Wnt7a to induce symmetric division.^7^^,^^43^ Contrastingly, asymmetric self-renewal results in one activated progenitor cell and another daughter cell destined for quiescence. Interestingly, MuSCs’ decision to undergo asymmetric or symmetric self-renewal is partially driven by the upregulation of fibronectin, an extracellular matrix glycoprotein ligand for the Syndecan-4/Frizzled-7 receptor complex (symmetric division promoter system).^7^ Fibronectin has been shown to be hypersecreted in MuSC-derived myotubes compared with quiescent MuSCs. Thus, when asymmetric self-renewal has satisfactorily replenished the myocyte population, MuSCs detect the increased extracellular fibronectin and begin symmetric self-renewal to replenish the stem cell niche. Another self-renewal pathway involves the protein sal003, an inhibitor of eukaryotic initiation factor 2α phosphatase. Sal003 prevents the phosphorylation of eukaryotic initiation factor 2α and promotes MuSC self-renewal.^87^ Through these 2 self-renewal methods and their regulation, the MuSC niche is constantly replenished. Assessment of possible deregulation and disruption of this intricate regulation of the MuSC niche and self-renewal program is warranted for understanding of the sequelae of rotator cuff injuries and the behavior of MuSCs secondary to an RCT in humans.

Similar to the self-renewal of MuSCs, differentiation into mature phenotypes also involves several distinct proteins and pathways. As discussed previously, upregulation of FGF instigates MuSC proliferation; conversely, upregulation of the natural FGF inhibitor, *Spry1*, leads to a return to quiescence and homeostasis of the MuSC pool.^68^ As expected, the Notch signaling pathway serves to regulate stem cell differentiation and thus MuSCs as well. The Notch intracellular domain was shown to regulate enhancers proximal to the collagen V (COLV) genes, leading to their upregulation.^4^^,^^84^ Consequently, the newly produced COLV interact with MuSCs through the calcitonin receptor which has been shown to delay the proliferation of MuSCs. ^4^^,^^84^ Thus, MuSCs can potentially be artificially maintained in quiescence through the Notch-COLV-calcitonin receptor signaling pathway. Another pathway controlling MuSC differentiation was discovered by Otto et al,^57^ connecting the Wnt signaling pathway to MuSC differentiation by showing that Wnt signaling may upregulate Pax7 and MyoD, thereby initiating MuSC proliferation.^59^ Immune cells can also regulate muscle stem cell populations through an inflammatory environment. For example, the presence of T cells, through the classic inflammatory pathway in which T cells produce cytokines such as IL-1α, IL-13, INF-γ, and TNF-α resulted in proliferation of MuSCs in a mouse model of muscle degeneration.^20^ As can be seen from the aforementioned examples, the regulation of MuSC proliferation is highly complex with multiple competing pathways. Therefore, it has been difficult to ascertain and pinpoint a particular pathway or singular protein responsible for activation of quiescent MuSCs. Thus, any adjuvant therapy may require a cocktail of various MuSC proliferative protein activators to yield an appreciable response.

## MuSC “niche” and adipogenesis

Muscle fatty infiltration of the supraspinatus and infraspinatus muscles after RCTs directly correlates with poorer outcomes and high retear rates after rotator cuff repair surgery.^18^ While the current understanding of the biologic mechanisms governing fatty infiltration of muscle after rotator cuff injury remains limited, the extracellular environment may be a contributing source. A possible cellular origin of adipocytes seen in rotator cuff fatty infiltration is PDGFRα^+^ progenitors as demonstrated in murine models.^45^^,^^47^^,^^71^ As with MuSCs, the regulation of PDGFRα^+^ progenitors is highly heterogeneous. Proinflammatory M1 macrophages secrete iNOS, TNF-α, and IL-12 inflammatory cytokines, which reduce adipogenic differentiation of PDGFRα^+^ preadipocytes via inhibition of adipogenic-related transcription factor expression.^47^ Conversely, recruited M2 macrophages, typically promoters of wound healing and fibrosis, express high levels of osteopontin that induces PDGFRα^+^ progenitor migration, proliferation, and differentiation via the CD44 receptor in a mouse model of fat remodeling.^45^

**Figure 3:**
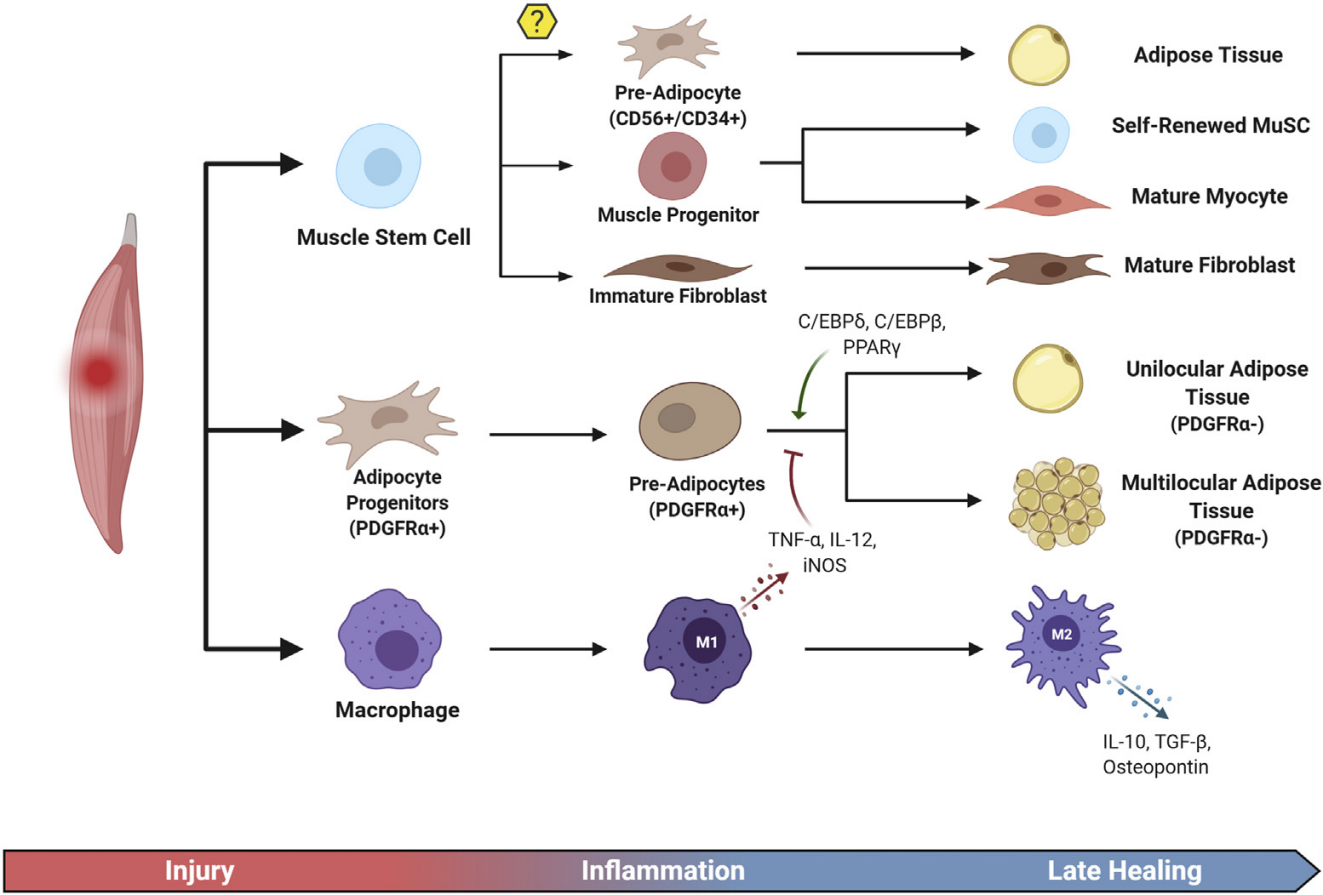
Summary of the proposed sources contributing to muscle fatty infiltration.

Currently, there is no consensus in the literature as to whether PDGFRα^+^ progenitors are the sole source of adipogenesis in skeletal muscle. MuSCs may also be a major contributor or auxiliary source of these adipocytes. To analyze MuSC differentiation pathways, some cellular markers must first be described. One of these markers is CD56 which is a human marker of MuSC-derived cells. CD56^+^ cells were once thought to be committed to the myogenic pathway.^12^ However, recent studies have shown that CD56^+^ cells are heterogeneous and differentiate into not only myotubes but also adipocytes (Fig. 3).^60^ Specifically, regarding adipogenesis from these MuSCs, Pisani et al^60^ isolated CD56^+^ cells and found that further sorting by CD34 could differentiate between those with adipogenic (CD34^+^) and myogenic (CD34^-^) potential. After acute injury, MuSCs become activated and have reduced expression of CD34, freeing the cell from CD34’s adhesive function to facilitate migration and ultimately promoting MuSCs proliferation at early stages of muscle regeneration.^1^ However, global knockout of CD34 in mice leads to defective muscle regeneration after injury.^1^ In summary, the PDGFRα^+^ progenitor cells, which may be a source of the fatty infiltration seen in rotator cuff tears, represent a distinct entity compared with the CD56^+^ myogenic cells.^78^ Location of PDGFRα+ vs. MuSCs may also help to explain which cellular populations are preferentially activated under certain conditions. PDGFRα+ progenitor cells are found in the interstitial space of mouse skeletal muscle, whereas CD56^+^ cells localize beneath the basal lamina.^78^ The combination of location and regulatory pathways differences in CD56^+^ myogenic cells and PDGFRα+ progenitor cells may shed light on the interplay of these 2 opposing forces in muscle regeneration vs. fatty degeneration.

## Isolation and transplantation of MuSCs

Before any adjuvant treatment methods can be developed, researchers must first be able to isolate MuSCs both *in vitro* and *in vivo*. The sheer variety of isolation techniques for human MuSCs – ranging from preplate technique to fluorescent-activated cell sorting (FACS) – in addition to an ill-defined set of cell surface markers, has made the isolation of MuSCs disorganized and inexact. Regardless of shifting definitions, there are some markers which represent promising starting points to solving the puzzle of MuSC isolation. For instance, myogenic MuSCs are negative for both CD45 (hematopoietic marker) and CD31 (endothelial marker).^23^^,^^54^^,^^85^ In contrast, MuSCs upregulate and are positive for Pax7, CXCR4, CD56, and CD29.^23^^,^^49^^,^^50^^,^^72^^,^^85^ This unique marker profile has allowed for researchers to fluorescently tag MuSCs and sort or identify them through FACS with varying success. The downside of FACS is that the populations of isolated MuSCs are typically small. The preplate technique attempts to overcome this problem by amplifying MuSC cell counts by performing cell culture after FACS. As with many *in vitro* techniques, the process of growing MuSCs outside of their native environment poses the risk of altered morphology, functionality, and gene expression.

Even with inexact isolation protocols, researchers have already attempted to transplant MuSCs to ameliorate wound healing.^23^^,^^85^ These transplanted MuSCs have been shown to retain a remarkable amount of plasticity in the target tissue while maintaining 2 core principles of stem cells – differentiation and self-renewal.^42^^,^^64^ First, transplanted MuSCs demonstrate the ability to proliferate and differentiate into myocytes to combat musculoskeletal degeneration with newly formed myofibers.^23^^,^^85^ Second, transplanted MuSCs also maintain the capacity to self-renew, allowing for the restoration of depleted MuSC niches in target organisms.^64^

## Therapeutic approaches via MuSCs

**Figure 4:**
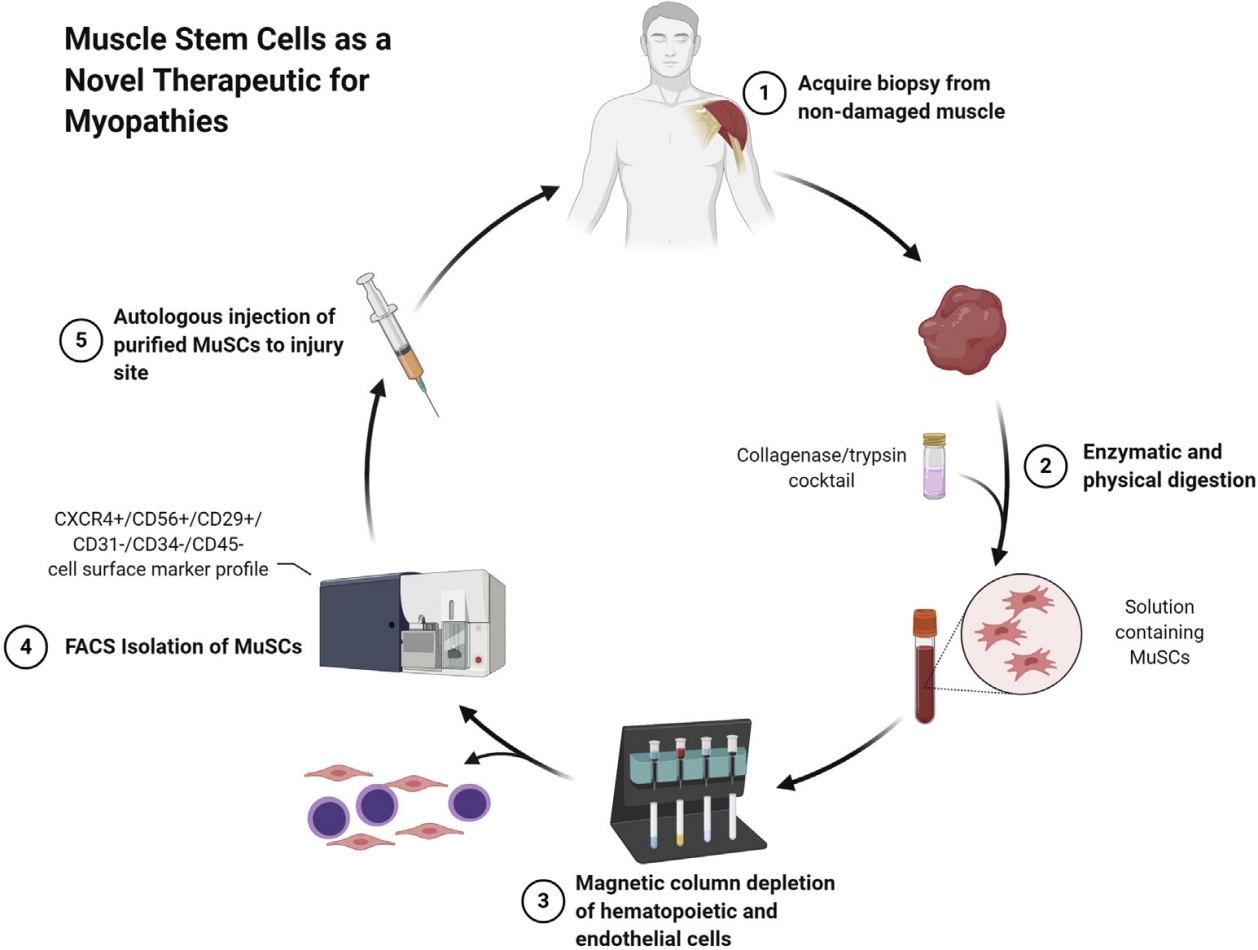
Schematic of muscle tissue processing and MuSC isolation for therapeutic application of MuSCs for treatment of myopathies. *MuSC*, muscle stem cell.

Cell transplantation as a biologic augmentation for rotator cuff repair has recently been demonstrated in the literature using mesenchymal stem cells.^29^^,^^38^ Hernigou et al^29^ observed that in forty-five matched patients who received mesenchymal stem cell adjunctive therapy vs. isolated rotator cuff repair alone, those with adjuvant mesenchymal stem cells had faster healing of the repaired cuff surface and fewer recurrent tears at the ten-year follow-up; a higher number of transplanted cells also correlated with a lower rate of loss of tendon integrity. Kim et al^38^ reported on 35 matched patients and found that injection of mesenchymal stem cells loaded in fibrin glue also decreased retear rates after arthroscopic rotator cuff repair, though there was no difference in pain, range of motion, or functional outcome measures at the 2-year minimum clinical follow-up. However, owing to the relative novelty of MuSCs, transplantations of MuSCs specifically have only been performed in murine models; thus, the histologic results and clinical consequences of MuSC transplantations in humans remain speculative. If human transplantation were to be considered in the future, as per our current understanding of the adipogenic vs. myogenic potential of these cells, transplanted cells should be CXCR4^+^/CD56^+^/CD29^+^/CD31^-^/CD34^-^/CD45^-^ (Fig. 4).^23^^,^^85^ Increased attention is currently being given to translating MuSC transplantation results from mice studies to human applications.^6^

Modulating the *in vivo* muscle stem cell and progenitor microenvironment in the rotator cuff to promote a myogenic environment affords an additional, less-invasive therapeutic strategy. Shirasawa et al^71^ designed a mouse model to recapitulate important characteristics of human muscular fatty infiltration after RCT and found rapid expansion of PDFGRα^+^ progenitors associated with subsequent adipocyte differentiation. Interestingly, treatment with oral imatinib mesylate led to decrease in PDFGRα^+^ cells, correlating with the attenuation of fatty infiltration. Imatinib also has an antifibrotic effect.^31^ On the other hand, fibroblast growth factor revitalizes the mitogenic milieu to support effective myogenesis.^69^ Gigliotti et al^25^ found that in human supraspinatus muscle tissue collected during cuff repair, the nitric oxide–donor drug isosorbide dinitrate induced a significant increase in MuSCs despite atrophy and possible denervation. Thus, proproliferative strategies such as isosorbide dinitrate may be particularly applicable in the aftermath of rotator cuff tears, specifically full-thickness supraspinatus tears, which have a reduced density of MuSCs and proliferating cells compared with partial-thickness tears.^48^ However, partial tears may also benefit given the fact that MuSCs demonstrate reduced proliferative capacity in partial-thickness tears compared with notear or complete-tear specimens, despite the fact that the pool of MuSCs is largest in partial tears.^54^

## MuSC misconceptions

It remains critically important to differentiate MuSCs and pluripotent embryonic stem cells. Embryonic stem cells are characterized by their rapid differentiation into multiple cell lineages with no tissue specificity, while MuSCs remain quiescent until they are activated by tissue damage or microenvironmental niche changes.^55^^,^^68^ Ergo, MuSCs operate in a less-restrictive niche than committed precursors but are not as differentially fluid as embryonic stem cells – existing between the two.

Second, there are often meaningful differences between rodent models and human morphology; this trend seems to hold true for MuSCs.^10^ For example, Oncostatin M has been shown to induce MuSC proliferation into myotubes in human cells.^65^ Contrastingly, in mice, Oncostatin M exposure preserves MuSCs in their quiescent niche.^41^ This diametrically opposite protein functionality suggests that a murine model may not be an adequate or accurate model organism for MuSC research if the final goal is human applications, owing to significant physiological and biochemical differences. This is underscored by the fact that mice undergo a different wound healing pathway as they possess a thin layer of musculature called the *panniculus carnosus* that provides a contractile potential to their skin, allowing large wounds to heal by contraction.^24^ Even more importantly, in rats with significant RCTs, there has been evidence of limited fatty degeneration when compared with humans.^27^ Given these differences between murine and human models of musculoskeletal degeneration, it appears that MuSC research should preferentially be performed in humans/human tissues to generate clinically relevant findings.

## Future directions and conclusions

While human MuSCs have been shown to have impaired proliferation in partial RCTs,^54^ very little is known about human MuSC precise role in RCT healing and pathophysiology. Moreover, while MuSCs have been well-characterized in murine models, this knowledge base is difficult to directly extrapolate to humans as MuSC physiology is quite species-specific.^10^ Therefore, further studies regarding the relationship of human MuSCs to muscle repair may open new avenues for therapeutic treatment of neuromuscular diseases via transplantation or forced differentiation. In particular, MuSC protein activators could be manipulated to aid in myofiber regeneration after massive RCT. Furthermore, as niche conditions in skeletal muscle influence stem cell proliferation, specific growth factor dosing, for example IGF1, could prime or encourage expansion and differentiation of skeletal muscle progenitors for increased muscle healing.^77^ However, owing to the sheer variety of proteins and pathways, there may not be a singular panacea, meaning that several differentiation pathways need to be altered simultaneously. Similarly, MuSC transplants could be introduced in the near future to improve postsurgical RCT retear rates. This adjuvant treatment method is not entirely outside the realm of possibility as hematopoietic stem cells are commonly used for treatment of leukemia and other similar diseases, and bone marrow aspirate concentrate is used to augment bone healing in fracture surgery.^28^^,^^30^ Regardless of implementation method, MuSCs represent a novel and potentially powerful adjuvant to improve the treatment and outcomes of massive RCTs.

## Disclaimers

Funding: No funding was disclosed by the author(s).

Conflicts of interest: The authors, their immediate families, and any research foundations with which they are affiliated have not received any financial payments or other benefits from any commercial entity related to the subject of this article.
